# Epstein-Barr virus-associated post-transplant lymphoproliferative disorders: beyond chemotherapy treatment

**DOI:** 10.20517/cdr.2021.34

**Published:** 2021-06-06

**Authors:** Sanam Shahid, Susan E. Prockop

**Affiliations:** Department of Pediatrics, Memorial Sloan Kettering Cancer Center, New York, NY 10065, USA.

**Keywords:** Post-transplant lymphoproliferative disease, PTLD, Epstein-Barr virus, EBV, rituximab, CD20 monoclonal antibody, immunotherapy, chemoimmunotherapy, EBV-specific cytotoxic T lymphocytes, EBV CTLs

## Abstract

Post-transplant lymphoproliferative disorder (PTLD) is a rare but life-threatening complication of both allogeneic solid organ (SOT) and hematopoietic cell transplantation (HCT). The histology of PTLD ranges from benign polyclonal lymphoproliferation to a lesion indistinguishable from classic monoclonal lymphoma. Most commonly, PTLDs are Epstein-Barr virus (EBV) positive and result from loss of immune surveillance over EBV. Treatment for PTLD differs from the treatment for typical non-Hodgkin lymphoma because prognostic factors are different, resistance to treatment is unique, and there are specific concerns for organ toxicity. While recipients of HCT have a limited time during which they are at risk for this complication, recipients of SOT have a lifelong requirement for immunosuppression, so approaches that limit compromising or help restore immune surveillance are of high interest. Furthermore, while EBV-positive and EBV-negative PTLDs are not intrinsically resistant to chemotherapy, the poor tolerance of chemotherapy in the post-transplant setting makes it essential to minimize potential treatment-related toxicities and explore alternative treatment algorithms. Therefore, reduced-toxicity approaches such as single-agent CD20 monoclonal antibodies or bortezomib, reduced dosing of standard chemotherapeutic agents, and non-chemotherapy-based approaches such as cytotoxic T cells have all been explored. Here, we review the chemotherapy and non-chemotherapy treatment landscape for PTLD.

## INTRODUCTION

Post-transplant lymphoproliferative disorder (PTLD) is a life-threatening complication of allogeneic solid organ (SOT) and hematopoietic cell (HCT) transplantation. PTLD encompasses a range of histopathology of abnormal lymphoid proliferations that occur in the context of defective immune surveillance experienced during and after transplant^[1]^. In the SOT setting, PTLD is most often derived from recipient lymphoid cells, while PTLD following HCT is almost exclusively of donor origin^[2,3]^. After HCT and early after SOT, the majority of PTLDs are driven by latency programs of Epstein-Barr virus (EBV) and reflect the requirement for active immune surveillance of EBV. PTLD developing after HCT is usually a high-grade monomorphic diffuse large B-cell lymphoma (DLBCL). These EBV-positive PTLDs are characterized by EBV-driven immortalization of B cells (and rarely T cells). Protection from EBV-positive PTLD in immune competent individuals is maintained by surveillance by cytotoxic T lymphocytes (CTLs) recognizing EBV^[4]^. A significant amount of immunologic energy is required to control EBV as these CTLs circulate at high frequency in normal EBV-immune individuals^[5]^. In HCT recipients, the risk of PTLD resolves after reconstitution of donor-derived immunity, whereas in recipients of SOT long-term immunosuppression translates into a long-term risk of both EBV-positive and EBV-negative PTLD - ranging in histology from benign lymphoid hypertrophy to aggressive lymphoma. Overall, PTLD after SOT has a bimodal incidence with “early” PTLD developing within a year of transplantation and being nearly uniformly EBV-positive, and while there is a second peak of “late” more commonly EBV-negative PTLD five-to-ten years after transplant, it is increasingly evident that the risk of PTLD continues for as long as the patient remains on immunosuppression^[1,6]^. In both settings, the incidence of EBV-positive PTLD is relatively low but carries significant risk of morbidity and mortality with 2-year survival in the SOT setting as low as 50%^[6]^ and as low as 10% after failure to respond to initial therapy^[7,8]^, as discussed in more detail later in this review. Similarly, recipients of HCT with EBV-positive PTLD that is refractory to initial therapy have dismal survival chances with median overall survival (OS) of just 1.7 months^[9]^. Restoration of EBV-directed immunity to achieve durable control of EBV should be a goal of treatment in recipients of both SOT and HCT with EBV-positive PTLD.

Factors that contribute to the risk of developing PTLD include patient age, type of HCT or type of organ transplanted in SOT, type and dosage regimen of immunosuppressive drugs, and pre-transplant EBV serostatus of both donor and recipient^[10,11]^. The reported incidence of PTLD is between 2% and 20%, with a greater number of cases in patients who receive a SOT compared to HCT recipients^[12,13]^. The incidence of PTLD varies by the organ(s) transplanted: intestinal and multi-organ transplants (5%−20%), lung and heart transplants (2%−10%), and renal and liver transplants (1%−5%)^[14]^. Over the past two decades, the incidence of PTLD has increased, presumably due to an increased number of organ transplants performed, use of novel immunosuppressive therapies, and improved diagnosis^[15,16]^. Simultaneously, a decreased risk of developing “early” EBV-positive PTLD has been reported^[17]^. Factors contributing to this are improved targeting of immunosuppressive medications, stringent monitoring of EBV viral load, EBV prophylaxis, and pre-emptive EBV-directed therapy^[18]^. Identification of modifiable risk factors should aid in delineating better strategies for prevention and treatment of EBV-positive PTLD.

Although this review will focus on EBV-associated PTLD, there are notable differences between patients with EBV-positive PTLD and those with EBV-negative PTLD. It has been demonstrated that patients with EBV-positive PTLD are typically younger, closer to transplant, and have a poorer performance status than those with EBV-negative PTLD^[19]^. Additionally, the European PTLD Network has demonstrated that while the incidence of extra-nodal disease is not significantly different between the two cohorts, those with EBV-positive PTLD are more likely to have allograft involvement and less likely to have nodal involvement^[19]^.

While prognostic features in patients diagnosed with PTLD after SOT and HCT have been established, these are distinct from those established for immune competent patients with non-Hodgkin lymphoma (NHL). NHL staging and prognosis depends on the number and location of involved lymph nodes, extra-nodal involvement, and presence or absence of bulky disease^[20]^. In contrast, the prognosis of PTLD depends on both disease-specific features (morphologic subtype, EBV positivity, and time from transplant) as well as patient‐specific factors (age, EBV serostatus, co‐morbidities, and ability to tolerate tapering of immunosuppression and intensive therapy)^[9,21]^. Given that no uniform prognostic scoring system has been used across prior reports in patients with PTLD, it is difficult to estimate the survival chances for individual patients, but OS ranges from 25% to 92% based on risk factors^[22]^.

For SOT recipients, the international prognostic index (IPI) uses risk factors of age greater than 60 years, Ann Arbor stage greater than or equal to III, Eastern Cooperative Oncology Group performance status greater than or equal to 2, elevated lactate dehydrogenase (LDH), and more than one extra-nodal site of involvement^[23]^. It was demonstrated that baseline IPI was a highly significant independent prognostic factor for time to progression (TTP) and OS in recipients of SOT^[8]^. A nationwide French study constructed a prognostic score that classified patients with PTLD as being at low, moderate, high, or very-high risk for death based on five variables at diagnosis: age, serum creatinine, LDH, PTLD localization, and histology^[22]^. A retrospective analysis validated the French prognostic scoring system in an independent cohort of 54 recipients of kidney transplant and a total of 122 additional SOT recipients^[24]^. However, in this cohort, the French scoring system was not superior to the classic IPI score. Furthermore, the type of transplant is a baseline variable that has an impact on OS and disease progression, which is not included in the current scoring systems^[23]^. Inability to tolerate reduction in immunosuppression (RIS) and lack of response to rituximab have also been reported as poor prognostic markers^[8]^. Prospective validation of prognostic scoring is essential for effectively implementing risk-stratified therapies^[24]^.

For HCT recipients, older age, extra-nodal disease, acute graft-versus-host disease (GVHD), and inability to tolerate RIS have been associated with an inferior response to first-line rituximab therapy^[25]^. Factors associated with a poor response to EBV-specific CTLs (EBV-CTLs) after prior rituximab included prior receipt of multi-agent chemotherapy, extra-nodal disease, and greater than three sites of disease^[26,27]^.

Treatment strategies for PTLD uniquely need to balance curative intent with the potential for injury to the transplanted organ and thus can differ from treatment of NHL in immunocompetent hosts. Furthermore, treatment strategies for PTLD after HCT differ from PTLD after SOT. Several studies demonstrate that outcomes are superior in patients on exogenous immunosuppression if that immunosuppression can be safely reduced^[25,28–30]^. First-line treatment in recipients of HCT is rituximab given that these patients have a high risk of significant toxicity from multi-agent chemotherapy. Though there are a few case reports showing a curative potential with multi-agent chemotherapy^[31,32]^, in general standard multi-agent chemotherapy is not successful and poorly tolerated in HCT recipients^[33]^. In contrast, the first-line treatment in PTLD emerging after SOT is RIS along with rituximab and/or multi-agent chemotherapy. Importantly, the safety of RIS varies with the organ transplanted as does the safety and tolerability of specific chemotherapy agents. In both cohorts of patients, adoptive therapy with EBV-CTLs has been explored in a series of clinical trials.

**Figure 1.**
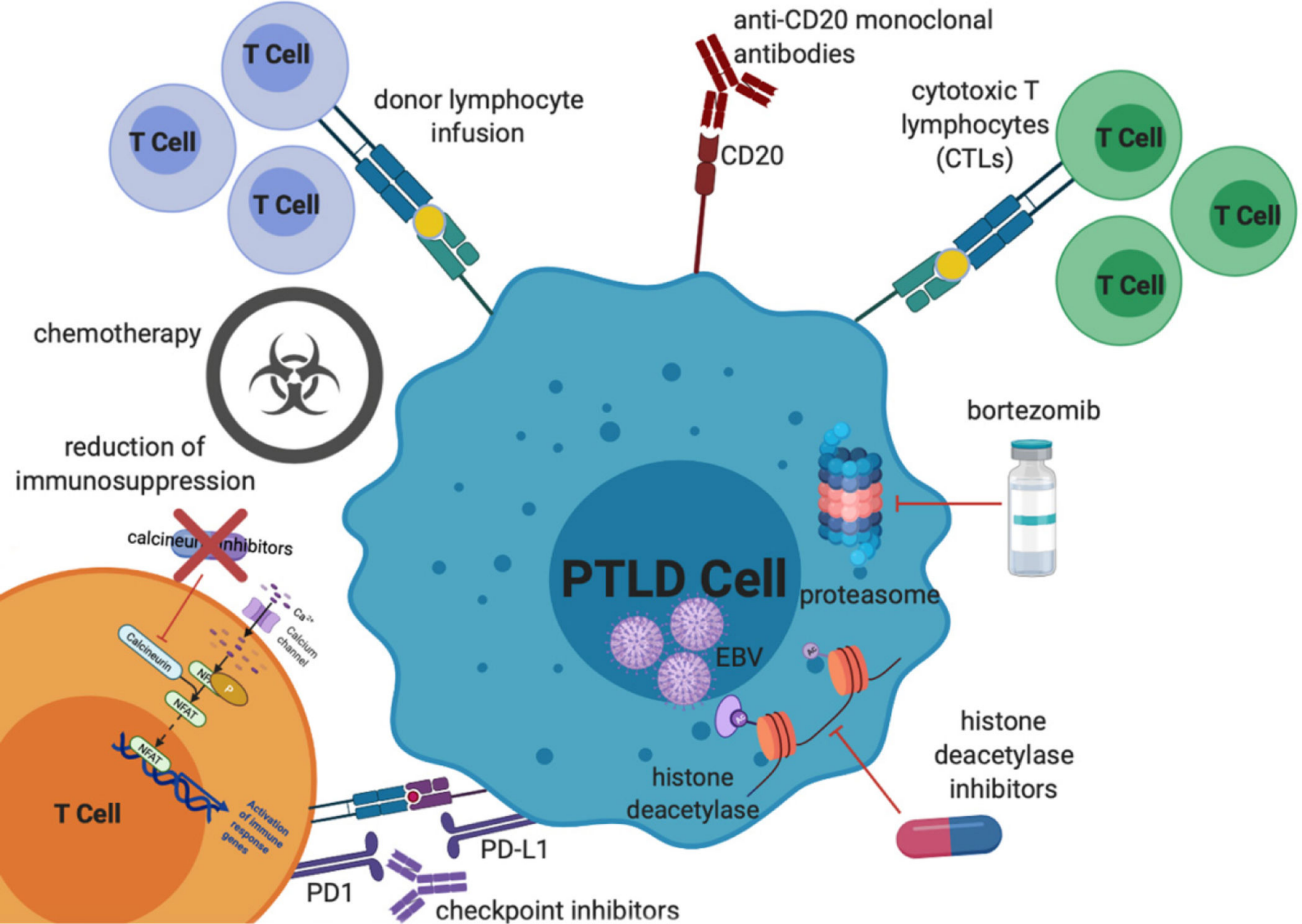
Mechanisms of treatment of EBV PTLD. Treatment options, which will all be discussed in further detail in this review, include reduction of immunosuppression, chemotherapy (including classical multi-agent lymphoma-based regimens as well as single agent anti-metabolite therapy), donor lymphocyte infusions, anti-CD20 monoclonal antibodies, cytotoxic T lymphocytes, proteasome inhibitors, histone deacetylase inhibitors, and checkpoint inhibitors. EBV: Epstein-Barr virus; PTLD: post-transplant lymphoproliferative disorder.

Here we review standard chemotherapy and non-chemotherapy approaches to treatment of EBV-positive PTLD [Figure 1], highlighting some of the unique aspects of treatment for this patient population.

## REDUCTION IN IMMUNOSUPPRESSION

The mainstay of first-line therapy for PTLD is RIS, which restores cellular immunity by re-establishing host T cell function to control lymphoproliferation^[30,34]^. Importantly, recipients of T-cell-depleted HCT are among those at highest risk after HCT and are not typically on exogenous immunosuppression. In addition, RIS may come at a cost of GVHD (HCT recipients) or transplant organ rejection (SOT recipients). The only prospective study evaluating RIS alone as treatment for PTLD in the SOT setting demonstrated a 6% partial response (PR) rate with no complete responses (CR)^[35]^. Furthermore, 38% of patients in this trial experienced graft rejection^[35]^. The published response rate to RIS ranges from 0% to 73%, and these responses are not typically sustained with durable responses maintained in less than 10% to 20% of cases^[36–42]^. Therefore, RIS is usually implemented during the initial workup of PTLD but then combined with other treatment modalities. These other treatment modalities, such as multi-agent chemotherapy or rituximab, impair host immunity and may protect from rejection in the context of RIS. Factors predictive of poor response to RIS in adult patients are EBV-negative disease, older age, elevated LDH, multi-organ involvement at diagnosis, bone marrow and liver involvement, B symptoms (fever, night sweats, and weight loss), severe organ dysfunction, dyspnea, and hepatitis C^[28,34]^. In pediatric SOT recipients, identified predictors for poor response to RIS include histologic features (CD20-negative, EBV-negative, Burkitt or Hodgkin morphology), late-onset disease, and central nervous system (CNS) involvement^[43–45]^.

Although limited, data demonstrate that immunosuppression with sirolimus may decrease the incidence of PTLD. In pre-clinical models of PTLD, high exposure to sirolimus has a potent anti-proliferative effect^[46]^. The published experience of this approach for treatment of PTLD is limited to three recipients of renal transplant described in two separate case series. In one, a patient was changed from tacrolimus and mycophenolate mofetil (MMF) to sirolimus and prednisolone and then experienced rapid improvement in allograft function and regression of the tumor that was durable at one year^[47]^. Similarly, two recipients of renal transplant with PTLD (DLBCL) had full regression of disease after switching from MMF or azathioprine to sirolimus^[48]^. Prospective clinical analysis is warranted to explore this further.

## CD20 MONOCLONAL ANTIBODIES

Rituximab, a first-generation chimeric monoclonal antibody targeting the B-cell surface protein CD20, has improved outcomes in many CD20-positive NHLs, such as DLBCL and follicular lymphoma. Rituximab is a standard component in the treatment of these CD20-positive lymphomas based upon a demonstrated survival benefit^[49]^. For recipients of HCT, American and European guidelines support pre-emptive treatment of EBV viremia with rituximab before the diagnosis of PTLD is established, while the role of preemptive treatment is less well established in SOT^[50,51]^. Rituximab is effective in EBV-positive PTLD because it directly targets the CD20-positive tumor. It may also improve disease control by residual EBV-CTLs by improving the effector to target cell ratio (E:T) of EBV-CTLs to EBV-infected B cells^[33]^. The major toxicities of rituximab include infusion reactions (e.g., fevers, rigors, and hypotension), cytopenias, infections related to immunosuppression, impaired humoral immune recovery, and prolonged hypogammaglobulinemia^[49]^.

A landmark retrospective review of PTLD emerging after HCT was performed by the European Group for Blood and Marrow Transplantation (EBMT)^[26]^. PTLD developed in 144 (3.22%) of 4446 transplants performed between 1999 and 2011, and survival was 69.4%^[26]^. Multivariable analysis revealed that a poor response to rituximab was associated with the following prognostic factors: age greater than or equal to 30 years, extra-nodal involvement, acute GVHD, and a failure to reduce immunosuppression at the time of diagnosis of PTLD^[26]^. PTLD mortality significantly increased with an increasing number of factors: zero to one, two, or three factors being associated with mortality of 7%, 37%, and 72%, respectively^[26]^. Both RIS at diagnosis and decreasing EBV viremia with initiation of therapy were predictive of an improved chance of survival^[26]^.

Single-agent rituximab achieves a CR in approximately 20% of patients with PTLD following either SOT or HCT^[8,36,52,53]^. Patients with CD20-positive PTLD who achieve a CR after rituximab induction therapy do not require chemotherapy^[8]^. However, they do remain at risk of recurrence. Single-agent rituximab was employed in a prospective multicenter phase II trial involving 43 evaluable patients with previously untreated CD20-positive PTLD that did not respond to tapering of immunosuppression^[54]^. The overall response rate (ORR) was 44%, while OS was 86% and 67% at 80 days and 1 year, respectively^[54]^. In another study, 60 SOT recipients with PTLD treated with single-agent rituximab had an ORR of 59% (42% CR)^[55]^. Half experienced disease progression within 6 months of completion of rituximab therapy^[55]^. Median OS was 35 months with 1- and 2-year OS rates of 73% and 52%, respectively^[55]^. In an international phase II trial, four additional courses of rituximab were administered to 37 of the 148 patients (25%) who achieved CR after four weekly doses of rituximab induction therapy for CD20-positive PTLD^[8]^. At 3 years, the estimated progression-free survival (PFS) and OS were 89% and 91%, respectively, among those who achieved CR after rituximab induction therapy^[8]^. In this trial, there was no significant difference in ORR, TTP, or OS between those with EBV-positive and EBV-negative PTLD^[8]^. Another prospective, multicenter, phase II trial in which PTLD patients who did not achieve a CR after RIS and four weekly infusions of rituximab then received a second course of four infusions revealed that extended treatment with rituximab can obtain a high rate of CR in patients with PTLD after SOT without increasing toxicity^[56]^.

Second and third generation CD20 monoclonal antibodies that decrease the murine components of rituximab include the fully human anti-CD20, ofatumumab (OFA), and the humanized anti-CD20, obinutuzumab (OBZ). Similar to rituximab, OFA is a type I anti-CD20 antibody and mediates efficacy by both complement- and Fc-mediated cytotoxicity^[57]^. OBZ, however, is a type II antibody and has limited complement-mediated cytotoxicity^[58]^. OFA is associated with a high frequency of infusion reactions, which are potentially fatal, and therefore requires steroid premedication^[59]^. OFA is also associated with more significant cytopenias. However, OFA is appropriate for patients with human anti-mouse antibodies (HAMA) or a history of serum sickness to rituximab^[60]^.

## CHEMOTHERAPY

The role of multi-agent chemotherapy for EBV-positive PTLD is very different in HCT *vs.* SOT patients and will be addressed separately.

In the treatment of EBV-positive PTLD emerging after HCT, multi-agent chemotherapy use has been limited to patients with a demonstrated first response to rituximab^[33]^. A retrospective study showed the limited efficacy of chemotherapy in patients who failed rituximab with no patient attaining CR^[31]^.

In SOT recipients, however, multi-agent chemotherapy has an established role for patients with CD20-positive PTLD^[61]^. Classically used regimens are rituximab, cyclophosphamide, doxorubicin, vincristine, and prednisone (R-CHOP) and dose-adjusted etoposide, prednisone, vincristine, cyclophosphamide, doxorubicin, and rituximab (EPOCH-R). There have been no randomized trials comparing different chemotherapy regimens in PTLD, and so physician experience and toxicity profile typically guide chemotherapy regimen selection. Furthermore, there are no trials that directly compare upfront chemoimmunotherapy to rituximab alone in CD20-positive PTLD.

Sequential treatment of CD20-positive PTLD (clinicaltrials.gov NCT01458548) established risk-stratified sequential treatment with four doses of rituximab with subsequent CHOP chemotherapy stratified based on response to rituximab. This is now becoming standard in the management of PTLD emerging after SOT and allows response to rituximab to be used as a prognostic factor for OS^[8]^. This group then hypothesized that rituximab consolidation might be sufficient treatment for patients with a CR after rituximab induction^[8]^. In a prospective, international, multicenter phase II trial, 152 treatment-naive adult SOT recipients with CD20-positive PTLD unresponsive to RIS were treated with four weekly doses of rituximab^[8]^. After restaging, complete responders received four courses of rituximab consolidation every 21 days; those not achieving a CR to induction rituximab received four courses of R-CHOP chemotherapy also in 21-day cycles^[8]^. One hundred and eleven of 126 patients had a complete or partial response (88%; 95%CI: 81%−93%), of whom 88 had a CR (70%; 95%CI: 61%−77%)^[8]^. Median OS was 6.6 years (95%CI: 5.5–7.6 years)^[8]^. The frequency of grade three or four infections and of treatment-related mortality was 34% (95%CI: 27%−42%) and 8% (95%CI: 5%−14%), respectively^[8]^. Response to rituximab induction remained a prognostic factor for OS despite treatment stratification^[8]^. This study concluded that in patients with PTLD, response to initial rituximab treatment can be used to stratify subsequent therapy into rituximab or R-CHOP consolidation and that this approach is feasible, safe, and effective.

An approach established in pediatrics in a phase II multicenter trial (clinicaltrials.gov NCT00066469) demonstrated safety and efficacy in combining low-dose cyclophosphamide and either prednisone or methylprednisolone with rituximab in pediatric and young adult patients who have CD20-positive EBV-positive PTLD following SOT^[62]^. Toxicity was similar for cycles of therapy with *vs.* without rituximab^[62]^. The CR rate was 69% (95%CI: 57%−84%)^[62]^. In addition, response appeared to improve after the completion of therapy: eight of 12 patients with radiographic evidence of persistent disease at the end of therapy achieved a CR by 28 weeks without further PTLD-directed therapy^[62]^. There were 10 deaths: three due to infections while receiving therapy and seven from PTLD^[62]^. The 2-year EFS was 71% (95%CI: 57%−82%) and OS was 83% (95%CI: 69%−91%)^[62]^. Based on these results, rituximab combined with low-dose chemotherapy has been adopted as first-line therapy by many clinicians for PTLD in pediatric recipients of SOT.

## OTHER AGENTS

Although currently not considered standard of care, other treatments such as bortezomib, bendamustine, and immune checkpoint inhibitors have been explored in the treatment of PTLD. Bortezomib, a proteasome inhibitor, is Food and Drug Administration (FDA)-approved to treat multiple myeloma and mantle cell lymphoma. It shows significant activity in lymphomas associated with EBV. A recent study (clinicaltrials.gov NCT01058239) in adult patients examined the effect of the addition of bortezomib to rituximab in the treatment of PTLD after SOT or HCT. A total of 7 patients were enrolled. ORR at 4 months was 42.9%, CR at 4 months was 42.9%, and PFS at 6 months was 43%. Adverse events were reported in all treated patients, for whom 28.6% were serious adverse events. Final results of this study are currently pending publication. Alternatively, bendamustine plus rituximab has demonstrated efficacy and a favorable toxicity profile in the treatment of mantle cell lymphoma and low-grade NHL^[63]^. There is a current phase II trial evaluating bendamustine combined with rituximab in patients with previously untreated PTLD (clinicaltrials.gov NCT02753062).

Microsatellite instability (MSI), due to mutations or deletions of genes involved in DNA mismatch repair, results in an accumulation of mutations. MSI has not been found in lymphomas developing in immune-competent individuals but has been reported in PTLD, unrelated to EBV status. In a series of 111 PTLD patient samples, 8.1% had microsatellite instability^[64]^. Programmed cell death protein 1 (PD-1) blockade has shown clinical benefit in MSI-high tumors after previous therapy^[65]^. Several series^[66–68]^ have demonstrated a potential role for the PD-1 /PD-L1 and PD-L2 pathway, including a nationwide study of 81 cases with 67% of PTLD diagnosed after SOT expressing PD-1 (on tumor-associated macrophages) and PD-L1/PD-L2 (on malignant cells)^[66]^. Gene expression profiling and analysis of copy number alteration (CNA) identified a prevalent CNA of the terminal region of chromosome 9 with expression data suggesting that PD-L2 is the target of this alteration^[67]^. However, there is limited experience in the treatment of PTLD with checkpoint blockade. A case report described control of EBV-positive PTLD in CNS with PD1 checkpoint blockade^[69]^. However, in preclinical mouse models PD-1 blockade aggravated the progression of EBV-positive PTLD^[70]^. Furthermore, there is a high potential risk of T-cell mediated allograft rejection and GVHD with the use of immune checkpoint inhibition in recipients of SOT and HCT.

Other novel agents currently in clinical trials for EBV-positive PTLD include nivolumab with EBV-CTLs (clinicaltrials.gov NCT02973113), brentuximab vedotin with rituximab (clinicaltrials.gov NCT01805037), everolimus with lenalidomide (clinicaltrials.gov NCT01075321), and everolimus with panobinostat (clinicaltrials.gov NCT00918333). Results of these studies will help elucidate the role of these novel therapies in EBV-positive PTLD.

## EBV-TARGETED THERAPIES

Typically, EBV-positive PTLD has a latency III EBV gene expression pattern^[71,72]^. In these tumors, EBV-induced B-cell transformation is sufficient to induce full malignant potential without secondary genetic or epigenetic changes^[71]^. These tumors are therefore different in pathogenesis from other EBV-positive malignancies where viral infection is the first event and then subsequent events such as c-myc translocations are required for malignant transformation^[68,71]^. For this reason, viral directed therapies are an appealing approach for PTLD.

### HDAC inhibitors combined with antivirals

Several antiviral compounds have been tested in the clinic against EBV-associated lymphomas, and in general, reproducible activity has been difficult to document^[73]^. Antivirals such as ganciclovir require lytic-phase proteins, including viral kinases (TK), to convert these drugs to active antivirals^[73]^. Thus, these drugs are not very effective at eliminating EBV in chronically infected hosts where the virus persists in a latent state. Induction of EBV TK within EBV-positive lymphomas is predicted to result in sensitization of these tumor cells to antiviral agents such as ganciclovir^[74]^. This approach is expected to have high tumor specificity based on targeting of EBV-containing cells.

The nucleoside analogs acyclovir and ganciclovir have activity against the EBV lytic phase TK, providing the rationale for attempts to induce expression of EBV lytic phase genes, including viral TK, as a way to sensitize EBV-infected targets to these agents^[75,76]^. As proof of concept, a cell line derived from a lung transplant recipient’s EBV-associated lymphoma was exposed to arginine butyrate, a histone deacetylase (HDAC) inhibitor, resulting in induction of EBV TK transcription, and culture in the presence of ganciclovir resulted in cell cycle arrest and cell death^[77]^.

This approach was explored in a Phase I/II study of arginine butyrate combined with ganciclovir. Fifteen patients with chemotherapy and/or radiation refractory EBV-positive PTLD were treated^[78]^. Antitumor activity was observed in 10 patients, with four CRs and six PRs. The most common toxicities were nausea and headache, the most common severe adverse events were related to the CNS, and rapid tumor lysis occurred in 3 patients.

Several other HDAC inhibitors can induce expression of EBV lytic phase genes *in vitro*, leading to the sensitization to nucleoside antivirals^[74,77]^. An actively recruiting Phase 1b/II study (clinicaltrials.gov NCT03397706) is designed to define a recommended Phase II dose of VRx-3996, an HDAC inhibitor, in combination with valganciclovir (Phase 1b) and to evaluate the efficacy of this combination in relapsed/refractory EBV-positive lymphomas (Phase II). There is also an active phase II trial of panobinostat, an HDAC inhibitor, monotherapy in patients with refractory lymphomas (clinicaltrials.gov NCT01261247).

### EBV cytotoxic lymphocytes

Adoptive cell therapy with EBV-CTLs or donor leukocyte infusion (DLI) transfers naturally occurring EBV-specific cytotoxic T cells that can kill EBV-transformed B cells into recipients with EBV-associated PTLD. This infusion of pathogen-specific T cells has been explored as a method to reconstitute viral-specific immune responses in the post-transplant period. Adoptive cell therapy relies on the fact that upon encountering a specific viral antigen presented by a specific and shared HLA allele, pathogen-specific T cells undergo *in vivo* expansion and mediate control of the infection. In contrast to chemotherapeutic approaches, adoptive cell therapy offers the potential to establish viral specific T cell memory while avoiding immune-ablation and organ toxicity^[79]^.

The initial demonstration that EBV-specific immunity can be transferred effectively was over 25 years ago in five recipients of T-cell-depleted allogeneic HCT who developed EBV-associated PTLD^[80]^. Patients received infusions of unirradiated DLI from their EBV-seropositive HCT donor, and all 5 patients had complete pathological or clinical responses^[80]^. This illustrated that unirradiated DLI was an effective source of EBV-specific T cell immunity for treatment for EBV-associated PTLD that arose after allogeneic HCT. However, it was associated with a significant risk of GVHD caused by the simultaneous transfer of alloreactive T cells.

Adoptive immunotherapy with EBV-CTLs generated from primary HCT donors is effective in the treatment of EBV disease complicating allogeneic HCT [Table 1]. EBV-specific CTLs have been effective in more than 80% of patients treated for overt PTLD^[81]^, a finding confirmed by other investigators in the treatment of transplant recipients with rituximab-resistant PTLD^[82–85]^. In SOT recipients, this approach is more challenging as the EBV-positive PTLD is typically of host origin and the organ donor is typically not HLA compatible and therefore not an appropriate source of EBV-CTLs. In addition, SOT recipients require long-term immunosuppression. Despite these challenges, several studies have demonstrated that autologous EBV-CTLs can be generated from recipients of SOT and when infused for treatment of EBV-positive PTLD, they can eradicate disease and restore EBV-specific immunity^[86–88]^.

The generation and use of donor-derived EBV-CTLs has thus far been explored on investigational protocols, but broader accessibility has been challenging. In addition to the specialized facilities required to generate cellular therapy products, other issues have limited accessibility of adoptive T cell therapy. These limitations have included the time (2–3 months) required to generate EBV-CTLs for adoptive therapy using the original methods. Given the rapid progression of disease in patients with EBV PTLD refractory to rituximab, donor-specific products would need to be generated before the onset of disease. Given the relatively low incidence of rituximab-refractory EBV-positive PTLD, this is not feasible.

One early approach to address these limitations was the use of non-toxic bridge therapy with agents such as hydroxyurea^[89]^. More recently, there have been two additional approaches to circumvent these limitations: one is development of techniques allowing the more rapid generation of EBV-CTLs and the second is the generation of banks of third-party EBV-CTLs available for immediate off-the-shelf use. A number of centers, and now commercial enterprises, have generated such banks of allogeneic EBV-CTLs with a published experience of 124 patients treated [Table 2].

The earliest demonstration of the potential safety of third-party EBV-CTLs was in 8 patients with EBV PTLD arising after SOT for whom autologous EBV-CTLs were not available^[90]^. This approach was expanded to a phase II clinical trial that treated 33 patients and resulted in a 64% response rate. In this study, recipients received EBV-CTL lines that were best matched for HLA defined at low resolution. Responses were better in those patients receiving EBV-CTLs that were matched at a higher number of HLA alleles and that had a higher fraction of CD4-positive T cells^[91]^. Another 5 patients treated with third-party EBV-CTLs resulted in four responders^[92,93]^, and ten pediatric SOT recipients produced an 80% overall remission rate^[94]^. A third-party, allogeneic, off-the-shelf bank of 330 GMP-grade EBV-CTL lines from specifically consented healthy EBV-seropositive HCT donors was used to treat 46 recipients of HCT (*n* = 33) or SOT (*n* = 13) with established EBV-positive PTLD, whose disease had failed to respond to or relapsed after rituximab therapy^[27]^. CR or sustained PR was achieved in 68% of HCT recipients and 54% of SOT recipients^[27]^. For patients who achieved CR/PR or stable disease after cycle one, one-year OS was 88.9%^[27]^ and 81.8%, respectively^[27]^. In addition, three of five recipients with progression of disease after a first cycle who received EBV-CTLs from a different donor achieved CR or durable PR (60%) and survived longer than one year. Maximal responses were achieved after a median of two cycles^[27]^. These results suggest that third-party EBV-CTLs should be further explored as therapy for rituximab-refractory EBV-positive PTLD emerging after either HCT or SOT.

Limited studies have demonstrated the durability of clinical responses to EBV-CTLs. A recent phase II clinical trial showed that both CR and PR to EBV-CTLs in SOT and HCT groups have been durable (6–115 months)^[27]^. In contrast, PRs with chemotherapy treatment typically are not durable. Although PRs to CTLs have been durable, there is no clinically validated definition for this assessment. For example, some institutions define a PR as a decrease in viral load by quantitative PCR of at least 50% from baseline or a 50% improvement in clinical signs and symptoms^[95,96]^. In contrast, other centers define a PR as a two-log decrease in viral load and resolution of symptoms^[27]^. There is a clear need for uniformity in disease assessment among clinicians.

The requirement for persistence of EBV-CTLs to maintain durable responses is also not defined. As long as 18^[97]^ and 24^[27]^ months after infusion of donor- or third-party-derived EBV-CTLs, it was possible to demonstrate the presence of CTL precursors responsive to either *in vivo* or *ex vivo* re-challenge. However, specialized techniques are required to demonstrate the origin of these precursors. Long-term follow-up of a cohort of patients treated with gene-marked donor-derived EBV-CTLs showed persistence of the EBV-CTLs even nine years after infusion^[98]^. In addition, persistence of viral-specific immunity may not depend on persistence of the infused populations. The development of epitope spreading beyond the initially targeted EBV antigens has been demonstrated with autologous products and may be critical for promoting and sustaining the antitumor response. This phenomenon implies a beneficial interaction between the infused populations and the tumor microenvironment and suggests that adoptive therapy with EBV-specific T cell therapy may potentiate reconstitution of EBV-specific immunity^[98]^. Therefore, persistence is not necessarily required for durability of response.

The primary safety concerns surrounding adoptive immunotherapy is GVHD in the HCT setting and allograft rejection in the SOT setting. As the frequency of circulating alloreactive T cells is similar, if not higher, than that of EBV-specific T cells in normal seropositive individuals, infusion of bulk donor lymphocytes carries a significant risk of GVHD or organ rejection. An evaluation of HCT donor-derived HLA-compatible DLI and HCT donor-derived HLA-compatible or disparate EBV-CTLs in 49 HCT recipients with biopsy-proven EBV-positive PTLD found reversible acute GVHD occurred in recipients of DLIs (17%) but not EBV-CTLs^[99]^. Overall, across studies of both donor-derived and third-party-derived EBV-CTLs, the incidence of GVHD has been limited^[86,88]^.

As a result of the efficacy and safety demonstrated to date, EBV-CTLs are considered an attractive therapeutic option and are included in the National Comprehensive Cancer Center (NCCN) guidelines for treatment of EBV-positive PTLD. While historically this approach was not broadly available, there are currently multicenter trials underway [Table 3] evaluating EBV-CTLs in rituximab-refractory EBV-positive PTLD, including at least one registrational trial (clinicaltrials.gov NCT03394365). While these trials are currently accruing without results available, the potential for an FDA-approved product is on the horizon.

Resistance to CTL therapy has been demonstrated to be contingent on multiple factors that are both recipient- and tumor-related and are important to define in order to improve upon experience to date. In the HLA disparate setting, it is important to ensure that partially HLA-matched EBV-CTLs are restricted by an HLA allele shared by the patient’s disease. Prior studies, both in mice bearing multiple EBV-BLCL xenografts^[100]^ and in patients receiving transplant donor-derived EBV-CTLs^[101]^, have shown that such T cells selectively accumulate in and only induce remissions of tumors co-expressing EBV and the HLA allele by which the EBV-CTLs are restricted. Since third-party EBV-CTLs are rarely fully HLA-matched, they may be restricted by HLA alleles not shared by the patient’s disease. Thus, selection on the basis of HLA restriction should supersede selection based on the number of shared HLA alleles. Additionally, mutations in tumor-specific antigens may lead to tumor escape as demonstrated by differential cytotoxicity against targets transformed with the endogenous strain of EBV compared to the B95.8 strain^[99]^. This was demonstrated in a patient where the major activity of the donor CTLs was directed against two HLA-A11-presented epitopes of the viral EBNA-3B antigen. Sequence analysis of the tumor revealed a deletion that removed both epitopes making it so that the tumor could not be recognized by the adoptively transferred EBV-CTLs^[102]^. Secondary treatment with EBV-CTLs restricted by a different HLA allele presenting a different vial epitope (called “switch therapy”) can overcome this event and induce remissions if initial EBV-CTLs are ineffective^[27]^. However, in addition to mutations of endogenous viral genes, the latency program expression by EBV-infected tumor cells is critical to the immunogenicity of EBV tumors and their recognition by adoptively transferred EBV-CTLs.

Despite advances in adoptive T cell therapy for EBV-positive lymphomas that express the full EBV latency III program, most non-PTLD EBV-positive lymphomas express the latency I and II programs, in which there are a more limited array of EBV encoded proteins presented - Epstein-Barr nuclear antigen (EBNA1) and latent membrane proteins (LMP 1 and 2), respectively. These programs are poorly immunogenic, enabling tumors to evade adoptively transferred T cells that more typically recognize the EBNA2 and 3 epitopes. In Burkitt lymphoma, progress is being made by identifying potent inducers of immunogenic EBV antigens such as the nucleic acid synthesis inhibitor, decitabine. Decitabine induces expression of the more immunogenic latent viral antigens expressed in EBV type II and III latency tumors, such as PTLD. Expression of these viral antigens in EBV I latency tumors, like Burkitt lymphoma, could improve the activity of virus-directed immunotherapies against these tumors^[103]^. In Hodgkin lymphoma and extranodal NK/T-cell lymphoma, which typically express the latency II program, treatment with autologous T cells directed to the LMP1 and/or LMP2 antigens has been reported as a safe and effective approach to induce durable complete responses without significant toxicity^[98,104,105]^.

One limitation to the durable efficacy of adoptively transferred EBV-CTLs in recipients of SOT may be the need for long-term immunosuppression to prevent allograft rejection in these patients. While durable responses have been seen in this patient population, strategies to protect adoptively transferred T cells from the immunosuppressive environment could improve on these results. One strategy is to genetically engineer EBV‐CTLs to be resistant to calcineurin inhibitors (cyclosporin A and tacrolimus), the most critical drugs used to prevent rejection after SOT and GVHD after HCT. Calcineurin inhibitors function by binding to cyclophilin (CyPA) and FK binding protein‐12 (FKBP‐12), respectively^[106]^. These complexes inhibit the calcium‐sensitive phosphatase calcineurin from binding to the transcription factor nuclear factor of activated T cells (NFAT) and prevent T cells activation^[106]^. To neutralize the immunosuppressive effects of these drugs, calcineurin mutants disrupt binding of tacrolimus-FKBP‐12 and/or cyclosporin-CyPA without affecting the active site responsible for NFAT dephosphorylation^[107]^. EBV‐CTLs expressing such mutants maintain their proliferative capacity and interferon‐γ secretion in response to stimulation with EBV^[107]^. An alternative way to protect adoptively transferred EBV-CTLs is to make them resistant to corticosteroids. Corticosteroid-resistant CTLs have also been developed but not yet applied to the setting of PTLD. Zinc finger nucleases that disrupt the glucocorticoid receptor gene in glioblastoma-specific CTLs prevent corticosteroid suppression of anti-tumor activity and permit the clinical use of an allogeneic cell product^[108]^.

### EBV cytotoxic lymphocytes combined with other therapies

There have been several attempts to combine EBV-CTLs with other agents in a multidisciplinary approach. For example, successful management of refractory EBV-associated PTLD, specifically DLBCL, with combined brentuximab vedotin and third-party EBV-CTLs has been described in a case report^[109]^. EBV-CTLs have also been combined with checkpoint therapy. Recent phase I/II trials have reported manageable toxicity profiles and promising anti-tumor activities of anti-PD-1 drugs (pembrolizumab, nivolumab, camrelizumab, and JS001) with/without chemotherapy in the treatment of recurrent/metastatic nasopharyngeal carcinoma^[110–112]^. While this approach is appealing, an anticipated limitation could be more rapid rejection of third-party CTLs and requires further clinical evaluation in a prospective setting.

### EBV-specific inhibitors

There is potential future use of EBV-specific inhibitors, including inhibitors of EBNA1 and EBNA2 as well as heat-shock protein 90 (HSP90) inhibitors. EBNA1 is consistently expressed in EBV-positive PTLD, and it is responsible for the attachment of the viral episome to human chromosomes and facilitates its segregation during cell division^[113]^. EBNA1-specific inhibitors that block the DNA-binding domain of EBNA1 and inhibit tumor growth *in vivo* have shown therapeutic potential^[113]^. EBNA2 is the first viral protein to be expressed upon EBV infection, and it is a potent inducer of viral (LMP1 and LMP2A) and cellular (c-myc, IL-18 receptor, and others) proteins^[114]^. EBNA2 inhibitors are therefore a potential therapeutic option for EBV-positive PTLD. HSP90 is induced in B cells early during EBV infection, expressed on the surface of EBV-transformed B cells, and upregulated on EBV-positive PTLD^[115]^. HSP90 inhibitors decrease *EBNA-1* and *LMP1* expression and translation leading to inhibition of growth in preclinical models^[115]^. All of these inhibitors are currently being evaluated in preclinical and clinical trials, but none are approved for clinical use at this time.

## CONCLUSION

Historically, management of PTLD has varied by recipient type (HCT *vs.* SOT), PTLD subtype, and treatment setting. There are practice differences based on whether patients are treated primarily by HCT or SOT transplant physicians, oncologists, or infectious disease specialists. Our understanding of risk stratification, treatment algorithms, and response will likely benefit from more uniform data collection and reporting as is currently being performed by some of the large registries for HCT and SOT recipients. Despite this landscape, there is increasing support for the use of single-agent rituximab as well as RIS as preemptive therapy for EBV viremia in high-risk patients, first-line therapy for PTLD in SOT recipients with risk-stratified sequential treatment, and second-line therapy in both HCT and SOT recipients with other treatments such as adoptive immunotherapy with EBV-CTLs. While the latter has been generally available only for those patients treated at selective sites or for patients willing and able to travel to such sites, this option is becoming more broadly available. A choice among therapies must take into consideration the aggressiveness of the PTLD, the expected time to response of individual therapies, and associated toxicities. Importantly, there is a clear need for uniformity in risk stratification of disease as well as in definitions of complete and partial responses and the timing of those assessments. Without more standardized approaches, it will be difficult to compare outcomes for the broadening array of therapeutic approaches. Furthermore, long-term studies are needed to evaluate toxicities and durability of responses to both chemotherapy and non-chemotherapy treatments such as CTLs. Overall, the future therapeutic perspective looks promising for PTLD with increasing availability of targeted chemotherapy and non-chemotherapy treatment options.

## Figures and Tables

**Table 1.** Experience using adoptive T-cell therapy for PTLD: donor-derived EBV CTLs

Study	Method of selection	Treatment setting	*N*	Response (CR + PR)	Acute GVHD
Rooney *et al.*^[116,117]^ Heslop *et al.*^[118]^	EBV-LCL sensitized	PTLD	13	11/13	8%
Lucas *et al.*^[119]^	EBV-LCL sensitized	PTLD	1	1/1	100%
Imashuku *et al.*^[120]^	EBV-LCL sensitized	PTLD	1	0/1	0%
Gottschalk *et al.*^[102]^	EBV-LCL sensitized	PTLD	1	0/1	Not documented
Comoli *et al.*^[121]^	EBV-LCL sensitized	PTLD	5	5/5	0%
Moosman *et al.*^[122]^	Peptide stimulation IFNγ capture	PTLD	6	3/6	0%
Doubrovina *et al.*^[99]^	EBV-LCL sensitized	PTLD	19	13/19	0%
Icheva *et al.*^[123]^	EBNA-1 peptide stimulation IFNγ capture	PTLD	8	6/8	13%
Gerdemann *et al.*^[124]^	DC nucleofection viral plasmids	PTLD	1	1/1	100%
Papadopoulou *et al.*^[96]^	Peptide-stimulated	PTLD	1	1/1	0%

PTLD: Post-transplant lymphoproliferative disorder; EBV CTLs: Epstein-Barr virus cytotoxic T cell lines; CR: complete responses; PR: partial response; GVHD: graft-versus-host disease.

**Table 2.** Experience using adoptive T-cell therapy for PTLD: third party EBV-CTLs

Study	Method of selection	Treatment setting	Prior therapy	*N*	HLA	Response (CR + PR)
Alabama Sun *et al.*^[93]^ Lucas *et al.*^[125]^	EBV-BLCL sensitized EBV-CTL	PTLD SOT	RT Rituximab/C	1 1	4/6 6/6	1/1 (100%) 1/1 (100%)
Edinburgh Haque *et al.*^[91]^	EBV-LCL sensitized EBV-CTL	PTLD HCT SOT	RIS Rituximab/C	2 31	2 – 5/6 2 – 5/6	2/2 (100%) 19/31 (61%)
Australia Gandhi *et al.*^[92]^	EBV-LCL sensitized EBV-CTL	PTLD SOT	RIS Rituximab/C	3	≥ 3/6	2/3 (66%)
Baylor Leen *et al.*^[126]^	Transduced multivirus	PTLD HCT	Rituximab	9	≥ 1	6/9 (67%)
Inserm Gallot *et al.*^[127]^	EBV-LCL sensitized EBV-CTL	PTLD HCT SOT	Rituximab/C Rituximab/C	6 3	≥ 2 ≥ 2	3/6 (50%) 1/3 (30%)
UK Chiou *et al.*^[94]^	EBV-LCL Stimulated EBV-CTL	PTLD SOT	Rituximab/RIS	10	Not specified	8/10 (80%)
Aberdeen Vickers *et al.*^[128]^	EBV-LCL Stimulated EBV-CTL	PTLD HCT SOT	N/A N/A	6 4	≥ 3 ≥ 3	4/6 (67%) 4/4 (80%)
Baylor Tzannou *et al.*^[95]^	Peptide stimulated	PTLD HCT	None	1	3/8	1/1 (100%)
MSK Prockop *et al.*^[27]^	EBV-LCL sensitized T-cell line	PTLD HCT SOT	Rituximab/C Rituximab/C	33 13	2–5/10 2–4/10	22/33 (68%) 7/13 (54%)
Spain Alonso *et al.*^[129]^	Not specified	PTLD HCT	Not documented	1	Not documented	0/1 (0%)

PTLD: Post-transplant lymphoproliferative disorder; EBV CTLs: Epstein-Barr virus cytotoxic T cell lines; CR: complete responses; PR: partial response.

**Table 3.** Currently recruiting EBV CTL trials for PTLD

Study	Intervention	Disease status
NCT03131934 Phase I	Tacrolimus-resistant autologous EBV CTLs	Newly diagnosed or rituximab refractory in SOT patients
NCT02779439 Phase I	HLA-matched third-party donor-derived specific CTLs	Viral infection following allogeneic HCT or SOT
NCT02900976 Phase II	Rituximab + LMP-specific T cells	Newly diagnosed, relapsed, or refractory in SOT patients
NCT03394365 Phase III	Tabelecleucel	(1) SOT after failure of rituximab and rituximab plus chemotherapy; or (2) allogeneic HCT after failure of rituximab
NCT02822495 Expanded access	Tabelecleucel	Relapsed/refractory in SOT and HCT patients

EBV CTLs: Epstein-Barr virus cytotoxic T cell lines; PTLD: post-transplant lymphoproliferative disorder; HCT: hematopoietic cell transplantation; LMP: latent membrane protein.
